# Construction and Evaluation of a Risk Score Model for Lymph Node Metastasis-Associated Circadian Clock Genes in Esophageal Squamous Carcinoma

**DOI:** 10.3390/cells11213432

**Published:** 2022-10-31

**Authors:** Jian Cheng, Fang Chen, Yufeng Cheng

**Affiliations:** 1 Department of Cancer Center, The Second Hospital of Shandong University, 247 Beiyuan Street, Jinan 250033, China; 2 Department of Pharmacy, The Second Hospital of Shandong University, 247 Beiyuan Street, Jinan 250033, China; 3 Department of Radiation Oncology, Qilu Hospital of Shandong University, No. 107 West Wenhua Road, Jinan 250012, China

**Keywords:** esophageal squamous carcinoma, differentially expressed lymph node metastasis-associated circadian clock genes, tumor microenvironment, prognosis, bioinformatics

## Abstract

Background: Studies suggested that circadian clock genes (CCGs) in human esophageal squamous carcinoma (ESCC) samples are dysregulated. However, the relevance of CCGs to lymph node metastasis (LNM) and prognosis of ESCC remains unclear. Methods: The differentially expressed genes (DEGs) between normal and ESCC samples in The Cancer Genome Atlas database (TCGA) database were intersected with the genes associated with LNM (LNMGs) in ESCC samples and 300 CCGs to obtain the differentially expressed LNM-associated CCGs (DE-LNM-CCGs). The risk model was constructed by Cox regression analysis in the TCGA-ESCC training set, and the accuracy of the risk model was verified by risk profile and overall survival profile. Furthermore, differences of 23 immune cells, 13 immune functions, and immune checkpoint molecules between the high- and low-risk groups were assessed using the single-sample gene set enrichment analysis (ssGSEA) algorithm. Gene set enrichment analysis (GSEA) was conducted to investigate the functional differences between low- and high-risk groups. Finally, we validated the mRNA expression levels of prognostic model genes by quantitative real-time polymerase chain reaction (qRT-PCR). Results: A total of six DE-LNM-CCGs were identified in TCGA-ESCC. TP53 and NAGLU were selected by Cox regression analysis to construct the risk model. Risk profile plots, overall survival plots, and validation results of the risk model in the validation set indicated that the constructed risk model was reliable. The result of ssGSEA showed that the percentages of activated B cells, activated dendritic cells, effector memory CD8 T cells, immune function in neutrophils, plasmacytoid dendritic cells, T cell co-inhibition, and Type 17 T helper cells were different between the high- and low-risk groups. In addition, the expression of CD274, PDCD1, TNFRSF18, and TNFRSF9 was dysregulated between the high- and low-risk groups. GSEA revealed that the high-risk group was associated with cell differentiation, oxidative phosphorylation, and steroid biosynthesis pathways, while the low-risk group was associated with chromosome, ECM–receptor interaction, and other pathways. Finally, qRT-PCR results showed that the mRNA expression levels of two prognostic genes were consistent with TCGA. Conclusion: In conclusion, the risk model constructed based on TP53 and NAGLU could accurately predict the prognosis.

## 1. Introduction

Esophageal cancer (EC) is the seventh most common malignancy and the sixth most common cause of cancer-related mortality worldwide [1]. EC includes two major pathological types: Esophageal squamous cell carcinoma (ESCC), and esophageal adenocarcinoma (EAC) [2]. ESCC accounts for 95% of all EC. Surgery is the main treatment method to remove EC, but the overall survival rate at 5 years after surgery is only 10% to 40% [3]. China accounts for approximately 50% of the incidence of global EC, of which roughly 90% is ESCC [4]. ESCC is characterized by early onset of lymph node metastasis (LNM) and LNM is a high-risk prognostic factor for ESCC [5]. Hence, it is essential to explore the relationship between ESCC and LNM and their underlying mechanisms to provide a reference for improving patient prognosis and providing new treatment strategies. 

Circadian clock genes (CCGs) are a series of genes that have periodic expression with the alternation of day and night [6]. Studies in human and animal models showed that cancer development was closely related to the loss of CCGs [7]. The BAML1 and PER families are representative of the CCGs [8]. The BMAL1 gene was found to reduce the occurrence and development of head and neck tumors by inhibiting the P13K-Akt-MMP-2 signaling pathway [9]. Other types of CCGs such as CLOCK1 and CRY1 were also [9] play important roles associated with breast cancer, prostate cancer, and other tumors [10]. Therefore, CCGs might provide new perspectives for cancer diagnosis and treatment. 

In this study, we identified NAGLU and TP53 as the key prognostic genes in ESCC through bioinformatics analysis, and a risk model constructed based on TP53 and NAGLU could accurately predict the prognosis of ESCC patients. Our findings might contribute to further understanding of the prognostic mechanism of ESCC and provide novel and valuable biomarkers to benefit patients with ESCC.

## 2. Material and Methods

### 2.1. Data Sources

The expression data of TCGA-ESCC were downloaded from The Cancer Genome Atlas (TCGA) database (https://portal.gdc.cancer.gov/, accessed on 4 January 2022). After excluding the samples of esophageal adenocarcinoma, a total of 99 samples including 11 normal and 88 ESCC samples were included in the study. In addition, we screened 46 ESCC samples with N0 stage as non-metastatic samples and 32 ESCC samples with N+ (N1\N2\N3) stage as LNM samples. Moreover, 300 CCGs were downloaded from the Molecular Signatures Database (MSigDB). Finally, samples with incomplete survival information and clinical information were excluded from all disease samples, and a total of 69 samples were used as a training set for risk model construction, survival analysis, and clinical correlation analysis. In addition, the GSE53622 dataset containing 60 ESCC samples with survival information was downloaded from the Gene Expression Omnibus (GEO) database as an external validation set.

### 2.2. Identification of Differentially Expressed Genes (DEGs) between ESCC and Normal Samples

To screen genes associated with ESCC, we performed differential analysis between normal samples (*n* = 11) and ESCC (*n* = 88) samples in the TCGA dataset using the “limma” R package (Vienna, Austria) [11], with the DEG screening condition of *p* < 0.05. [12], and used the “ggplot2” (version 3.3.5) [13] R package and the “pheatmap” (version 1.0.12) R package to draw a volcano map and a heat map to show DEG expression.

### 2.3. Identification of Differentially Expressed LNM-Associated CCGs (DE-LNM-CCGs) 

Differential analysis was performed using the “limma” R package [11] to obtain differences of CCGs between ESCC patients with LNM (*n* = 32) and without (*n* = 46) LNM. The screening condition was *p* < 0.05. Similarly, volcano and heat maps of LNMGs were drawn using the “ggplot2” (version 3.3.5) [13] and “pheatmap” (version 1.0.12) R packages. Finally, we took the intersection of DEGs, LNMGs, and 300 CCGs to obtain DE-LNM-CCGs and displayed them using a Venn diagram.

### 2.4. Construction of Risk Model 

To construct the DE-LNM-CCG-related prognostic signature, Cox regression analysis was performed on the training set. Univariate Cox regression analysis was selected to screen DE-LNM-CCGs associated with ESCC prognosis. Then, genes with *p* < 0.05 in the results of the univariate Cox regression were involved in the multivariate Cox regression analysis and the regression coefficients of the identified prognostic genes were calculated. Finally, the risk model was established based on the genes and corresponding coefficients acquired from the multivariate Cox regression analysis. Namely, the risk score was calculated using the predict.coxph function, and the formula was as follows:

$$\mathrm{Riskscore}\;=\;h0\left(t\right)\times \;\exp\left(\beta_{1}\beta 1\times 1+{\;\beta }_{2}X_{2}+\ldots +{\;\beta }_{n}X_{n}\right).$$

β refers to the regression coefficient, for which the HR value can be obtained after taking the inverse natural log exp(β); ho(t) is the baseline risk function; h(t, X) is the risk function associated with X (covariate) at time t. After multivariate Cox regression modeling, the value of the risk score we compute with the predict function is actually h(t, X). X1, X2 are the prognosis-related genes obtained by Cox analysis. Then the optimal threshold was screened using the surv_cutpoint function of the “survminer” R package, and samples in the training set were divided into high- and low-risk groups based on the optimal threshold.

### 2.5. Validation of the Prognostic Risk Model

To assess the validity of the risk model, a risk profile was plotted based on the risk score. The risk profile consists of three parts: risk curve, scatter plot, and expression heat map of the model genes. Kaplan–Meier (K-M) survival analysis was used to estimate survival differences between high- and low-risk groups. The receiver operating characteristic (ROC) curve is a graph that shows the effect of the classification model under all classification thresholds. The area of the lower part of the curve is called the area under the curve (AUC), which is used to indicate the prediction accuracy and sensitivity. Higher AUC values indicate higher prediction accuracy [14]. Based on the risk model obtained, the ROC curve was used to calculate the AUC area of the model to evaluate the validity of the model, and the ROC curve was plotted using the “survivalROC” R package with 1, 3, and 5 years as the survival time points. In addition, the risk model was validated using the GSE53622 validation set, and risk curves, scatter plots, expression heat maps of model genes in high- and low-risk groups, survival curves in high- and low-risk groups, and ROC curves were also plotted for the data from the validation set.

### 2.6. Risk Score and Clinical Trait Correlation Analysis

To more closely understand the correlation between clinical and risk scores of ESCC, we performed a stratified survival analysis of high- and low-risk groups across clinical characteristics. The impact of risk score and clinical factors (T-stage, N-stage, gender, age, stage, alcohol combination) on patient survival was then analyzed using the analysis of ROC curves.

### 2.7. Immune Infiltration Analysis

Tumor microenvironment (TME) cells are an important component of tumor tissue. A growing body of evidence elucidates their clinicopathological significance in predicting prognosis and treatment outcomes [15]. The percentages of immune cells and the expression level of associated genes of immune function and immune signalling pathway activity of each sample were obtained by the single-sample gene set enrichment analysis (ssGSEA) algorithm and then grouped according to immune activity. Differences between 23 immune cells and 13 immune functions in the high- and low-risk groups were compared using the Wilcoxon test, and violin and heat maps were drawn using the “ggplot2” R package and “pheatmap” (version 1.0.12) R package.

### 2.8. Analysis of Immune Check Loci in High- and Low-Risk Groups

We used the Wilcoxon test to compare the differences in the 24 immune check loci in the high-risk and low-risk groups. Next, the IMvigor210 dataset [16] was also used to determine the response to immunotherapy in the high- and low-risk groups, and the differences in the results were statistically tested by means of the chi-square test. *p* < 0.05 indicated significant difference.

### 2.9. Gene Set Enrichment Analysis (GSEA) Functional Enrichment Analysis for High- and Low-Risk Groups

To explore signaling pathways that were differentially activated associated with the high- and low-risk group samples from the TCGA database, we aligned an ordered list of genes by the “limma” package with log|FC| from highest to lowest. Then, we downloaded GO.v7.4 and GKEGG.v7.4 gene sets from MSigDB of Broad Institute and used the clusterProfiler parkage to perform GSEA on all genes in the high- and low-risk group samples in TCGA database to find the common functions and related pathways of a large number of genes within the gene sets. |NES| > 1, FDR < 0.25, NOM *p* < 0.05 were used as screening conditions.

### 2.10. Quantitative Real-Time Polymerase Chain Reaction (qRT-PCR) Validation

Normal esophageal epithelial cell lines (T-HEECs) as well as esophageal squamous carcinoma cell lines KYSE-30, KYSE-150, KYSE-410 were used for PCR to verify the expression of prognostic model genes. These cell lines were purchased from Saibaikang Biotechnology Co., Ltd. in Shanghai, China. T-HEECs and KYSE-30 were cultured in DMEM (Gibco, Shanghai, China) with 10% fetal bovine serum in a cell culture incubator at 37 °C with 5% CO2. KYSE-30 and KYSE-150 were cultured in RPMI 1640 medium (Gibco, China) in a cell culture incubator at 37 °C with 5% CO_2_. Total RNA was extracted from all cell lines with Trizol reagent (CAT.-G356281) provided by Ambion. Then, we used sweScript RT I First Strand cDNA Synthesis All-in-OneTM First-Strand cDNA Synthesis Kit (CAT.-G33330-50) provided by Servicebio (Wuhan, China) for reverse transcription reaction. PCR was performed using the 2xUniversal Blue SYBR Green qPCR Master Mix (CAT-G3326-05) kit provided by Servicebio. The PCR conditions were: 95 °C pre-denaturation for 1 min, and then 40 cycles. Each cycle included 95 °C denaturation for 20 s, 55 °C annealing for 20 s, and 72 °C extension for 30 s. GAPDH was used as an internal reference for gene detection. Primer sequences are shown in Table 1. Three biological replicates were used in this study. T-HEECs and the expression of the biomarkers in KYSE-30, KYSE-150, and KYSE-410 cell lines were compared by analysis of variance (ANOVA). *p* < 0.05 was considered a significant difference.

## 3. Results

### 3.1. Identification of DEGs between ESCC and Normal Samples

**Figure 1:**
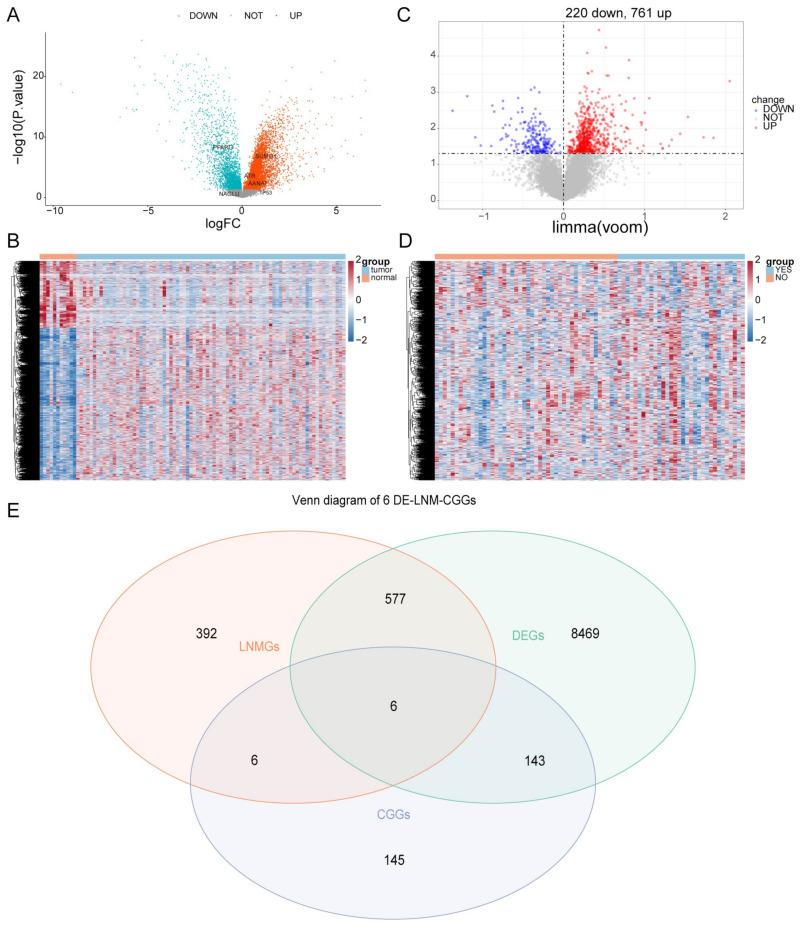
Differentially expressed CCGs in ESCC and normal samples. (**A**) The volcano map of CCGs. (**B**) The heat map of CCG expression levels. (**C**) The volcano map of differential genes in N1 and N0 samples. (**D**) The heat map of DEGs between N1 and N0 samples. (**E**) Venn diagram of LNMGs, CCGs, and the DEGS between ESCC and normal samples.

A total of 9195 genes were significantly differentially expressed in ESCC samples compared with normal samples, of which 6379 genes were upregulated and 2816 genes were downregulated (Supplementary Table S1), and the volcano and heat map results of DEGs are shown in Figure 1A,B.

### 3.2. Identification of DE-LNM-CCGs 

Differential analysis of patients with and without lymphatic metastasis in ESCC samples yielded LNMGs. The results of the differential analysis showed that there were 981 LNMGs in the sample, of which 761 were upregulated and 220 were downregulated. Figure 1C,D show the volcano and heat maps of differential genes between ESCC patients with and without LNM (Supplementary Table S2). A total of six DE-LNM-CCGs were obtained after crossover of LNMGs and CCGs with the DEGs of ESCC and normal samples, including ATR, AANAT, NAGLU, PPARG, TP53, SUMO1 (Figure 1E).

### 3.3. Construction of Risk Models

**Figure 2:**
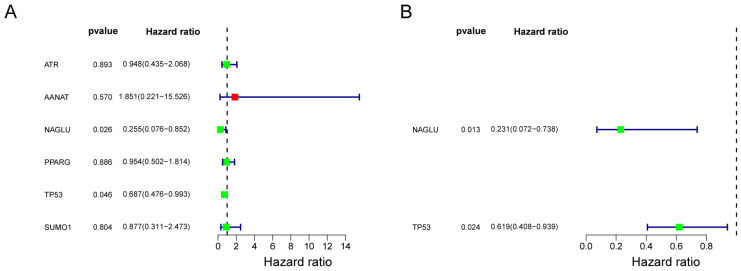
Risk model constructed with Cox regression analysis. (**A**) Forest plot for univariate analysis. (**B**) Forest plot for mutivariate Cox regression analysis. Green: Hazard ratio (HR) < 1; red: HR > 1.

The study constructed risk models by univariate Cox regression analysis and multivariate Cox regression analysis. The results of the univariate Cox analysis are shown in Figure 2A, with *p* < 0.05 for two genes, NAGLU and TP53 (Table 2). Multivariate Cox analysis finally showed that NAGLU and TP53 constructed the best model, and they were used as prognostic biomarkers for the construction of the risk model (Figure 2B), and the weighted gene expression levels of each of the two genes were summed with their respective coefficients to calculate the risk score:

$$\mathrm{Riskscore}\;=\;\mathrm{ho}\left(t\right)\times \;\exp\;\left(\mathrm{NAGLU}\;\times \left(-1.464\right)+\;\mathrm{TP}53\times \left(-0.4797\right)\right).$$

The 69 ESCC samples were divided into high- and low-risk groups with 42 and 27 samples in each group according to the optimal threshold for patients with ESCC in the training set.

### 3.4. Validation of the Prognostic Risk Model

**Figure 3:**
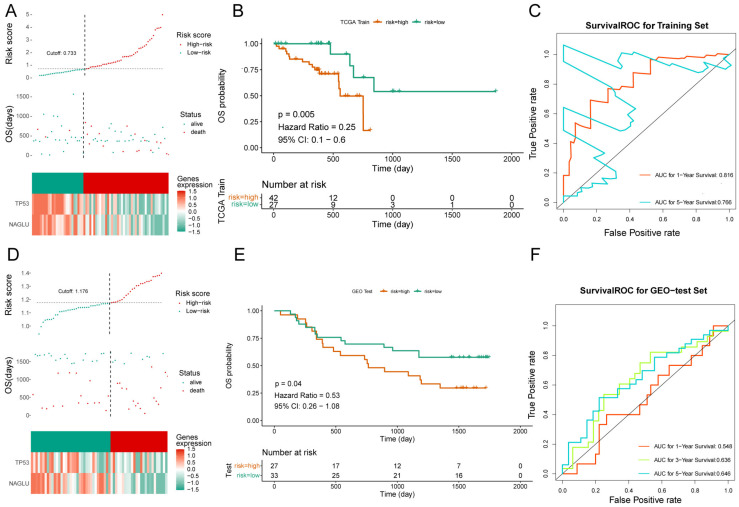
Risk model validation and evaluation. (**A**) Risk curve for high-risk subgroups in the training set. (**B**) Survival curve for high- and low-risk subgroups in the training set. (**C**) ROC curve of the training set. (**D**) Risk curve for high-risk subgroups in the validation set. (**E**) Survival curve for high- and low-risk subgroups in the validation set. (**F**) ROC curve of the validation set.

The risk curve plot consists of upper, middle, and lower plots, the horizontal coordinates are the samples of patients ranked according to their risk scores, the horizontal coordinates in the upper and middle plots are the same, the risk scores increase from left to right; the vertical coordinates are the risk scores and survival time, respectively, the dashed lines are the median risk scores and their corresponding numbers of patients, the lower plot is the heat map of the expression of model genes in the high- and low-risk groups (Figure 3A). K-M survival analysis showed that patients in the high-risk group in the training set had worse survival (Figure 3B, Supplementary Table S3). The results of the ROC curves are shown in Figure 3C. The 1-, 3-, and 5-year AUCs of the ROC curves in the training set were all greater than 0.76, indicating the better efficacy of the risk model.

The risk curves, scatter plots, and expression heat maps of model genes in the high- and low-risk groups in the validation set are shown in Figure 3D, and the results of survival curves and ROC curves in the high- and low-risk groups are shown in Figure 3E,F. It can be seen that the survival rate of the high-risk group in the validation set was low (Supplementary Table S4), and the AUCs of the 3-year and 5-year ROC curves were >0.63, indicating that the efficacy of the risk model in the validation set was better.

### 3.5. Risk Score and Clinical Trait Correlation Analysis

**Figure 4:**
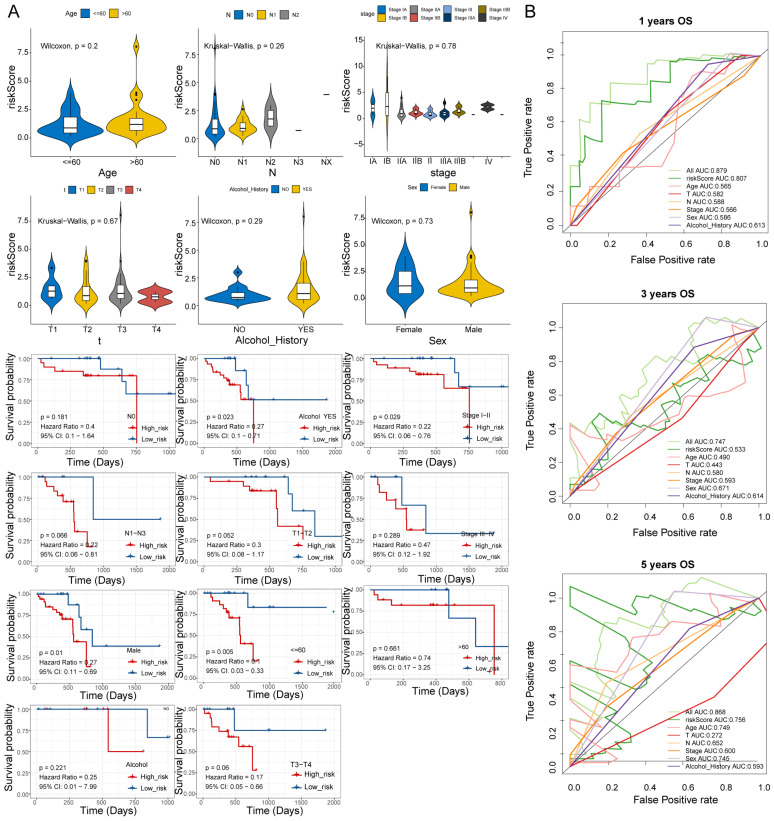
Correlation analysis of risk score and clinical traits. (**A**) Stratified survival analysis of risk scores and clinical traits. (**B**) ROC analysis of risk scores and clinical traits in patient survival.

Stratified survival analysis of risk scores and clinical traits analyzed the correlation between ESCC clinical and risk scores. The results of stratified survival analysis of high- and low-risk groups in different clinical traits showed significant differences in N1–N3, alcohol YES, and stage I–II subgroups in high- and low-risk groups (Figure 4A). Baseline demographic and clinical characteristics of the included patents are shown in Supplementary Table S5, and the results suggested that the high- and low-risk groups in the N1–N3 stage had significant differences. In addition, the ROC results of risk score and clinical traits in patient survival are shown in Figure 4B, where the best AUC curve values were obtained for the risk score and all clinical factors combined in the 1-year, 3-year, and 5-year overall survival ROC curves, reaching 0.879, 0.747, and 0.868, respectively.

### 3.6. Immune Infiltration Analysis

**Figure 5:**
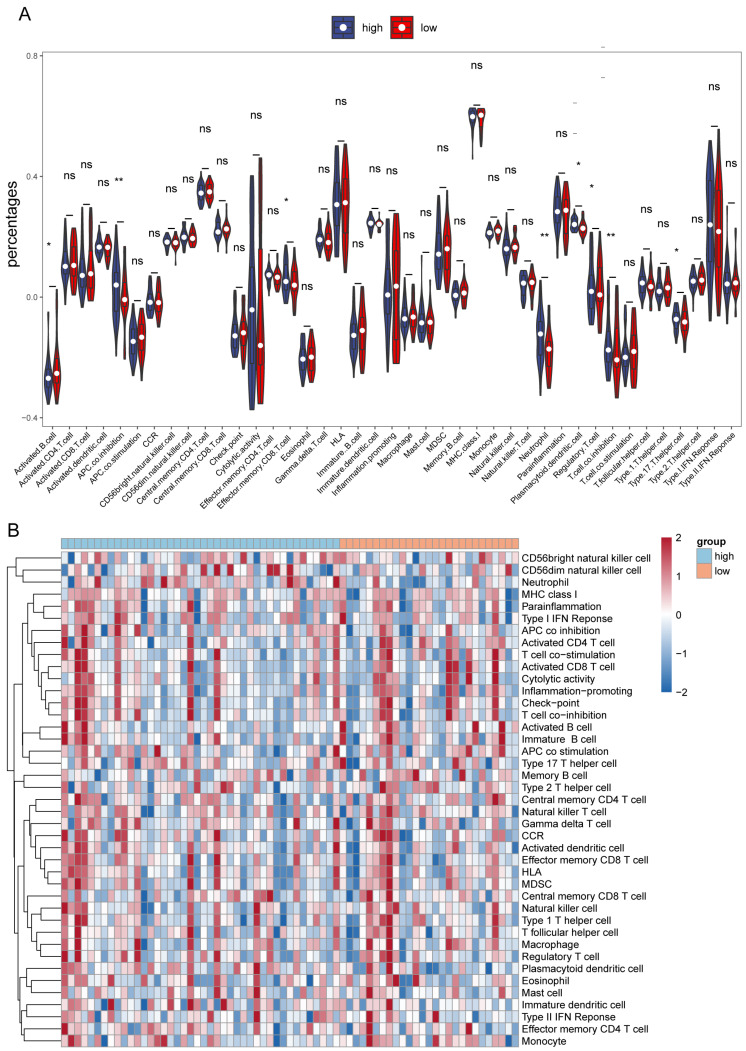
ssGSEA analysis in high- and low-risk groups. (**A**) The violin plot of immune scores of immune cell types in high- and low-risk groups. (**B**) Heat map of immune scores of immune cell types in high- and low-risk groups. * represents *p* < 0.05, ** represents *p*< 0.01, ns represents no significant difference.

The level of immune cell infiltration is important in predicting prognosis and treatment outcome. The study used the ssGSEA algorithm to assess differences in 23 immune cell types and 13 immune functions in high- and low-risk groups (Supplementary Table S6). The results of the violin plot and heat map of immune scores of immune cell types in high- and low-risk groups are shown in Figure 5A,B. The results indicated that there were differences in activated B cells, activated dendritic cells, and effector memory CD8 T cells among immune cells. Among the 13 immune functions, there were differences in neutrophils, plasmacytoid dendritic cells, T cell co-inhibition, and Type 17 T helper cells.

### 3.7. Analysis of Immune Check Loci in High- and Low-Risk Groups

**Figure 6:**
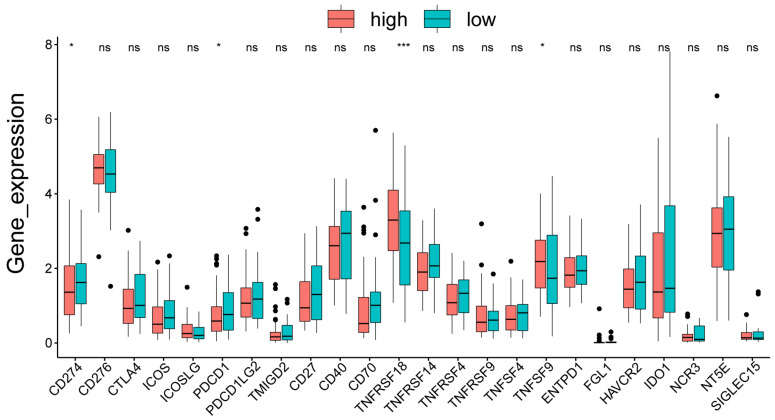
Analysis of immune check loci in high- and low-risk groups. * represents *p* < 0.05, *** represents *p* < 0.001, ns represents no significant difference.

The result of the differences in the four immune check loci in the high- and low-risk groups is shown in Figure 6, with significant differences in the CD274, PDCD1, TNFRSF18, and TNFRSF9 immune check loci.

### 3.8. GSEA Functional Enrichment Analysis for High- and Low-Risk Groups

**Figure 7:**
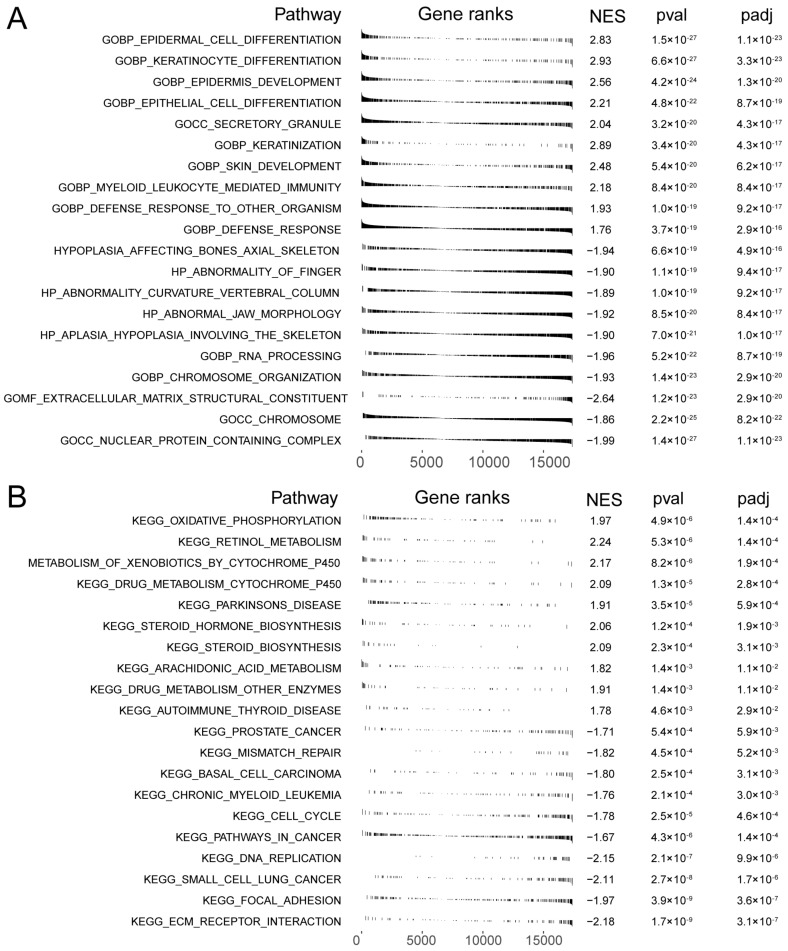
GSEA functional enrichment analysis in high- and low-risk groups. (**A**) Gene enrichment with GO terms of the selected genes. (**B**) KEGG enrichment analysis in high- and low-risk groups.

GSEA can search for common functions and related pathways of genes within the gene set. The results of Gene Ontology (GO) functional enrichment analysis in high- and low-risk groups showed that a total of 894 pathways were significantly enriched in the high-risk group and 2470 pathways were significantly enriched in the low-risk group. Figure 7A shows the top 10 pathways that were significantly enriched in the high- and low-risk groups, respectively, by GO enrichment results (Supplementary Table S7). The results showed that the pathways significantly enriched in the high-risk group are epidermal cell differentiation, keratinocyte differentiation, epidermal development, epithelial cell differentiation, keratinization, skin development, myeloid leukocyte-mediated immunity, and defense response to bacteria. Significantly enriched pathways in the low-risk group were nuclear protein complexes, chromosomes, extracellular matrix structural components, chromosome organization, and RNA processing.

The results of Kyoto Encyclopedia of Genes and Genomes (KEGG) functional enrichment analysis in the high- and low-risk groups showed that 23 pathways were significantly enriched in the high-risk group and 29 pathways were significantly enriched in the low-risk group. Figure 7B shows the top 10 pathways that were significantly enriched in the high- and low-risk groups, respectively (Supplementary Table S8). The results showed that the pathways significantly enriched in the high-risk group are oxidative phosphorylation, retinol metabolism, cytochrome p450 metabolism of xenobiotics, drug metabolism cytochrome p450, Parkinson’s disease, steroid hormone biosynthesis, steroid biosynthesis, arachidonic acid metabolism, and autoimmune thyroid disease. Pathways that were significant in the low-risk group were ECM–receptor interactions, adherent spots, small cell lung cancer, DNA replication, and cell cycle.

### 3.9. qPCR Validation

**Figure 8:**
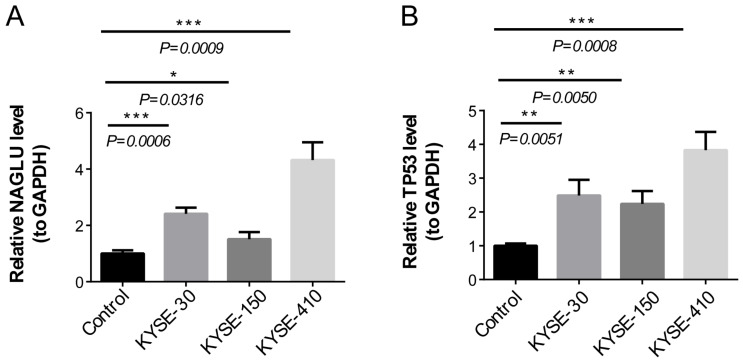
Comparison of differences in TP53 and NAGLU genes in KYSE-30, KYSE-150, and KYSE-410 cell lines. (**A**) The expression level of TP53 in normal cells (T-HEECs) and KYSE-30, KYSE-150, and KYSE-410 cell lines by qRT-PCR. (**B**) The expression level of NAGLU in normal cells (T-HEECs) and KYSE-30, KYSE-150, and KYSE-410 cell lines by qRT-PCR. * represents *p* < 0.05, ** represents *p* < 0.01, *** represents *p* < 0.001, and ns represents no significant difference.

To further validate the biomarker expression, we used qRT-PCR to compare the expression levels of TP53 and NAGLU genes in normal cells (T-HEECs) and KYSE-30, KYSE-150, and KYSE-410 cell lines. The results suggested the expression of TP53 and NAGLU genes in KYSE-30, KYSE-150, and KYSE-410 cell lines was significantly higher than in normal cells (T-HEECs) (Figure 8).

## 4. Discussion

Increasing evidence suggested that tumorigenesis was clearly affected by circadian mechanisms [17]. Previous studies found that CCGs in ESSC patients can serve as independent prognostic biomarkers [18]. LNM is also a high-risk prognostic factor for ESCC. Reliable and accurate prediction of prognostic biomarkers in ESSC is important for determining treatment options and improving prognosis. Therefore, this study explored the relevance of DE-LNM-CCGs to the prognosis of patients with ESCC based on bioinformatics.

In this study, a risk model was constructed based on TP53 and NAGLU, and the risk model was able to accurately predict the prognosis of ESCC patients. The results showed that patients in the high-risk group had a lower survival rate and a poorer prognosis, and that the risk model we developed was reliable. Chen et al. used bioinformatics methods to identify ESCC methylation-driven genes and established a reliable risk prognosis model consisting of GPBAR1, OLFM4, FOXI2, and CASP10 and it provided potential biomarkers for the early treatment and prognosis evaluation of ESCC [19]. Zhang and colleagues reported that the 6-gene-related risk score prognostic model based on these genes, including TSPAN2, AMBP, ITLN1, C6, PRLR, and MADCAM1, may be a reliable tool for predicting the prognosis of patients with ESCC [20]. Compared with previous studies, our model was constructed based on DE-LNM-CCGs. The results suggested that the model based on TP53 and NAGLU had a predictable prognosis for ESCC patients. To the best of our knowledge, TP53- and NAGLU-related prognostic models have not been reported in previous studies.

In this study, we identified two prognostic genes, TP53 and NAGLU. Previous studies suggested that TP53 is the most frequently mutated gene across several tumors, especially advanced metastatic disease [21]. Yao and colleagues found that TP53 might be the dominant factor promoting ESCC pathogenesis and had prognostic value in ESCC [22]. Zhang and colleagues established a gene signature based on TP53 mutation status, which is an independent prognostic factor in ESCC patients and showed promising sensitivity and specificity for survival predictions [23]. These results are consistent with our findings, pointing out that TP53 has a positive correlation with ESCC patients’ prognosis. The majority of all human tumors have mutations in the TP53 gene, which encodes the p53 protein. Research showed that p53 acts as a transcription factor that regulates the circadian clock by direct control of Per2 expression [24]. The NAGLU gene encoding α-N-acetylglucosaminidase is located on chromosome 17q21.1. NAGLU contains six exons, and the cDNA coding for a 720 amino acid protein [25]. Previous studies have shown that deficiency of NAGLU mainly causes several human disease states, including mucopolysaccharidosis ⅢB and atherosclerosis [26,27]. Notably, our findings suggest that NAGLU might have the potential to serve as a prognostic gene for ESCC. At present, the mechanism of NAGLU in ESCC has not been fully elucidated. Further studies are warranted to illuminate the prognostic value of NAGLU and its relevant mechanisms in patients with ESCC.

The composition of immunocytes in the tumor microenvironment is also known to affect cancer prognosis [28]. ssGSEA analysis demonstrated that the immune state was significantly different between the low-risk and high-risk ESCC patients, including the activated B cells, activated dendritic cells, and effector memory CD8 T cells. A previous study found that patients with ESCC with high CD8 density after neoadjuvant chemoradiation therapy were significantly associated with better survival [29]. Our result was also consistent with the data of a previous study showing that infiltration of CD8+T cells can be used adjunctively with PD-L1 expression as a predictive marker for nivolumab treatment in ESCC [30]. However, the prognostic effect of CD8+T cells needs to be confirmed by further experimental and clinical exploration. In addition, there are not enough data available to show the presence of the activated B cells and activated dendritic cells in human ESCC tissues, and their roles in the antitumor immune reaction. Interestingly, we found an important phenomenon in which the expression of immune checkpoint genes CD274, PDCD1, TNFRSF18, and TNFRSF9 in normal esophageal tissues was time-dependent in the database. These results suggest a possible link between the circadian clock and immune infiltration.

In addition, KEGG and GO enrichment analysis showed that the high-risk group was associated with cell differentiation, oxidative phosphorylation, and steroid biosynthesis pathways, while the low-risk group was associated with chromosome, ECM–receptor interaction, and other pathways. A study showed that the oxidative phosphorylation signaling pathway was important in the occurrence and progression of ESCC [31]. Li et al. demonstrated that cloperastine inhibits the proliferation of ESCC by suppressing mitochondrial oxidative phosphorylation [32]. In addition, a number of studies revealed that aberrant activation of the ECM–receptor interaction signal pathway is related to tumorigenesis of multiple human malignancies, such as hepatocellular [33], colorectal [34], and breast cancer [35]. Until now, very little was known about the ECM–receptor interaction pathway in ESCC.

Nonetheless, there are still some limitations in this study. First, although we performed qPCR analysis, the role and mechanism of prognostic genes associated with ESCC need to be explored deeply. Second, although high-risk and low-risk analysis has been carried out, ideally time series studies with a defined cohort that has multiple time-interval samples should be performed to check for the activation/inhibition of the signaling factors identified. As there is a lack of such datasets in public databases, such research cannot be carried out. In conclusion, our study identified DE-LNM-CCGs that can be used as prognostic biomarkers for ESCC and have important clinical implications for the diagnosis of ESCC patients.

## Figures and Tables

**Table 1:** The sequences of primers.

Gene Name	Primer Sequence	
	Forward Primer (5′-3′)	Reverse Primer (5′-3′)
TP53	TTGATTCCACACCCCCG	CGCCTCACAACCTCCGT
NAGLU	TTGCTGAGTTCTTCGGTG	CGCTTGTGGGAGATTTTC
GAPDH	CCCATCACCATCTTCCAGG	CATCACGCCACAGTTTCCC

**Table 2:** Descriptions of CCGs of the risk score model in Cox proportional hazard regression analysis.

ID	HR	HR.95L	HR.95H	*p*-Value
ATR	0.947993233	0.43463116	2.067709939	0.893222348
AANAT	1.850888642	0.220651998	15.52575458	0.570468921
NAGLU	0.25525297	0.076453577	0.852204455	0.026420866
PPARG	0.953966365	0.501622693	1.814215819	0.885738052
TP53	0.687444889	0.475704346	0.993433169	0.046038893
SUMO1	0.876880467	0.310946046	2.4728385	0.803839818

ATR: ATR Serine/Threonine Kinase; AANAT: Aralkylamine N-Acetyltransferase; NAGLU: N-Acetyl-Alpha-Glucosaminidase; PPARG: Peroxisome Proliferator Activated Receptor Gamma; TP53: Tumor Protein P53; SUMO1: Small Ubiquitin Like Modifier 1.

## Data Availability

The datasets generated and analyzed during the current study are included in the article.

## Supplementary Materials

The following supporting information can be downloaded at https://www.mdpi.com/article/10.3390/cells11213432/s1, Table S1. Differentially expressed of CCGsin ESCC and normal samples. Table S2. Differential genes in N1 and N0 samples. Table S3. Survival curve for high- and low-risk subgroups in the training set. Table S4. Survival curve for high- and low-risk subgroups in the validation set. Table S5. Baseline demographic and clinical characteristics of the included patents. Table S6. Differences in 23 immune cell types and 13 immune functions in high- and low-risk groups. Table S7. The top10 pathways that were significantly enriched in the high and low risk groups. Table S8. The top 10 pathways that were significantly enriched in the high and low risk groups.
